# Left atrial volume assessed by echocardiography identifies patients with high risk of adverse outcome after acute myocardial infarction

**DOI:** 10.1186/s44156-024-00060-1

**Published:** 2024-10-21

**Authors:** Jorun Tangen, Thuy Mi Nguyen, Daniela Melichova, Lars Gunnar Klaeboe, Marianne Forsa, Kristoffer Andresen, Adrien Al Wazzan, Oyvind Lie, Fatih Kizilaslan, Kristina Haugaa, Helge Skulstad, Harald Brunvand, Thor Edvardsen

**Affiliations:** 1 ProCardio Center for Innovation, Department of Cardiology, Oslo University Hospital, Rikshospitalet Sognsvannsveien 20, Nydalen, PO Box 4950, Oslo, NO-0424 Norway; 2 Institute for Clinical Medicine, University of Oslo, Sognsvannsveien 9, Oslo, 0373 Norway; 3 Department of Cardiology, Hospital of Southern Norway, Sykehusveien 1, Arendal, 4838 Norway; 4 Oslo Centre for Biostatistics and Epidemiology, Department of Biostatistics, University of Oslo, Oslo, Norway

**Keywords:** Acute myocardial infarction, New treatment strategies, Echocardiography, Indexed left atrial volume, Primary outcomes

## Abstract

**Background:**

The left atrial (LA) volume has been demonstrated to be an important predictor of adverse outcome in patients with various cardiac conditions, including acute myocardial infarction (AMI). However, new treatment strategies in patients with AMI have led to better patient outcomes. We hypothesised that increased LA size could still predict mortality in patients with AMI despite improved treatment strategies.

**Methods:**

We included patients with AMI in a prospective multicenter cohort study and the study patients were enrolled from 2014 to 2022. We recorded echocardiographic and clinical data during their index hospitalisation. Indexed LA volume (LAVi) was assessed in all patients and was used as a continuous variable in the univariate and multivariate Cox regression analysis. The study took place over a period of five years and median follow-up time was 3.8 years (range 3.1 to 5.0 years). The primary study outcomes were all-cause mortality and major adverse cardiac events (MACE). MACE was defined as hospital readmission due to myocardial infarction, cardiac arrest, stroke, heart failure, or onset of new atrial fibrillation.

**Results:**

We included 487 patients (69 ± 12 years old, 26% female) with AMI. During the follow-up period all-cause mortality was 50 (10.3%) and patients who reached the primary outcomes were 153 (31.4%). The deceased patients had higher LAVi compared to survivors (40.0 ± 12.9 mL/m^2^ vs. 29.7 ± 11.2 mL/m^2^, *p* < 0.001). Factors associated with all-cause mortality and MACE were age, year of enrollment, left ventricular (LV) ejection fraction, LV global longitudinal strain (GLS), LV filling pressure, moderate or severe mitral regurgitation and LAVi. GLS and EF were segregated into two distinct models due to their moderately high correlation (*r* = 0.57, *p* < 0.001). LAVi remained as an independent echocardiographic predictor of primary outcomes after adjusting for the covariates above in two separates multivariable Cox regression models (hazard ratio 1.02/1.02 mL/m^2^ [95% CI 1.01–1.03/1.01–1.03], *p* = 0.006/0.003).

**Conclusions:**

Our study demonstrated that LA dilatation is an independent echocardiographic predictor of mortality and MACE in patients with AMI despite improved treatment strategies. This finding highlights the potential of using LAVi as a marker for prognostication in these patients.

**Supplementary Information:**

The online version contains supplementary material available at 10.1186/s44156-024-00060-1.

## Background

Previous studies have demonstrated that left atrial (LA) volume serves as a robust predictor of overall mortality after acute myocardial infarction (AMI) where an enlarged LA is indicative of unfavorable prognosis among patients with AMI [1–3].

The LA is directly linked to the left ventricle (LV) through the open mitral valve during ventricular diastole. Consequently, the LA volume is affected by the same factors that influence diastolic LV filling. [4] In contrast to Doppler variables of LV diastolic function, which can be influenced by acute hemodynamic changes, the LA volume reflects the duration and severity of diastolic dysfunction. [5] Notably, LA volume integrates the impact of elevated LV filling pressures as a consequence of preexisting cardiovascular conditions and acute diseases. As a result, it has emerged as a valuable indicator of cardiovascular risk. [6]

Recent updates in guidelines and the implementation of novel treatment strategies have yielded improved prognosis in patients with AMI. [7, 8] Modern treatment strategies with early use of angiography and percutaneous coronary intervention (PCI) during the initial hospital stay, [7, 9] duel antiplatelet therapy (DAPT) [10–12] and treatment with drug-eluting stents (DES) instead of bare-metal stents (BMS) [8, 13–16] have shown to reduce the mortality over time. In light of these advancements, our hypothesis was that LA volume retains its significance as a predictor of mortality and major adverse cardiac events (MACE) even after the adoption of new and updated treatment guidelines for AMI patients.

## Methods

### Study population

We included patients with AMI in this prospective multicenter cohort study at three hospitals in Norway (Hospital of Southern of Norway, Arendal and Kristiansand and Oslo University Hospital, Rikshospitalet) over a period of five years. The study patients were enrolled from 2014 to 2022 and were part of a prospective, observational, multicenter, follow-up study of the Norwegian IMPROVE study (clin trials: NCT02286908) with aim to explore the predictive value of strain echocardiography in risk prediction of patients with heart diseases.

The study patients were treated according to current guidelines [7, 8] and the study did not interfere with acute treatment. The study included patients post-PCI on the day of their hospital discharge. There were no in-hospital deaths. Informed consent was obtained from all patients and the study was approved by the local ethics committee (REK 2013/573). Myocardial infarction was verified to a combination of criteria required to meet the diagnosis of AMI. [7, 8] We excluded patients with type 2 infarction and other causes of chest pain.

Patients were revascularised with PCI in 92% of the cases and the rest of the patients were treated medicinally only (8%). We included 453 (93%) patients with non-ST-elevation myocardial infarction and 34 (7%) with ST-elevation myocardial infarction. Baseline demographic data and presenting features were recorded upon admission. Clinical signs of HF at admission were recorded using New York Heart Association Functional Classification. High-sensitivity cardiac troponin I or T was taken on admission and was above the 99th percentile of the upper reference limit (> 45 ng/L or > 14 ng/L respectively) in all our patients. Medications at discharge were recorded. All patients were followed till the end of the study or until they died.

### Echocardiography

All patients underwent transthoracic echocardiography after invasive procedures and before discharge (within 48 h after myocardial infarction) using Vivid E9/E95 (GE, Vingmed, Horten, Norway). All studies were performed by experienced sonographers and reviewed by staff cardiologists with advanced echocardiography training. The data were digitally recorded for analysis using EchoPac version 203/204 software from GE Healthcare, Vingmed.

LV volume and ejection fraction (EF) were measured by the biplane modified Simpson´s method with ranges and severity of cut-off values for LV EF according to guidelines. [17] A total of 11 patients were excluded from the study due to poor image quality of GLS, EF, and/or LA volume measurements.

GLS was calculated as average of segmental peak systolic myocardial strain from the three standard apical views by two-dimensional speckle tracking echocardiography. [18] All calculations with GLS in the present study are reported with an absolute value for simplicity.

LA volume was assessed by the biplane area-length method from apical 4- and 2-chamber views. Measurements were obtained in end systole and indexed for body surface area. [19] The LA volume was measured from the best possible non-foreshortened view of the 4-chamber view. We used three cardiac cycles per view and chose the best cycle for further analysis. LA dilatation was defined as LAVi > 34 mL/m^2^. [17, 20] LA volume was used as a continuous variable in the univariate and the multivariate Cox regression analysis.

LV filling pressure was evaluated according to the 2022 European Association of Cardiovascular Imaging consensus document, [19] using a combination of five echocardiographic parameters including LA reservoir strain. [21] This algorithm integrates E/A, E/e’, tricuspid regurgitation velocity, LAVi and LA reservoir strain, each with defined cut-off values. LA reservoir strain was the latest addition to this algorithm and was analyzed using a dedicated LA automated functional imaging software. [22] LA reservoir strain was utilised as a binary variable and strain < 18% was consistent with elevated LV filling pressure (Additional file 1). [21] In our AF patients (*n* = 27), the evaluation of LV filling pressure was performed by averaging parameters over multiple heartbeats. Criteria such as septal E/e’ >11, E-deceleration time ≤ 160 ms, isovolumic relaxation time ≤ 65 ms, and elevated tricuspid regurgitation velocity > 2.8 m/s (in the absence of pulmonary disease) were indicative of elevated LV filling pressure. [19]

The severity of mitral regurgitation (MR) was semi-quantitatively graded based on the color-flow Doppler data and by measuring the width of the vena contracta [23]. We dichotomised MR in absent/mild or moderate/severe regurgitation. [23, 24]

### Grading coronary artery lesions and treatment

Coronary lesions were graded by visual assessment of angiographic stenosis where > 50% diameter reduction was considered significant. Coronary artery disease was defined in a wide spectrum from no significant stenosis to complex multivessel (≥ two vessel). Patients were revascularised with PCI. Those patients who were not revascularised had no significant stenosis or were not suitable for PCI and had medical therapy only.

### Outcome variables

The primary clinical outcomes assessed in this study encompassed all-cause mortality and MACE. MACE was defined as hospital readmission due to myocardial infarction, cardiac arrest, stroke, heart failure (HF), and/or the onset of new atrial fibrillation (AF).

### Statistical analysis

Continuous variables were expressed as mean ± SD and compared using an unpaired t test when data were normally distributed or median as Interquartile Range and the Mann-Whitney U test when data were not normally distributed. Categorical data were presented as number and percentage and were compared using a chi-square or fisher´s exact test. A two-sided p-value of < 0.05 was considered statistically significant. All statistical analyses were performed using Stata software version 17.0 (StataCorp 4905 Lakeway Drive College Station, Texas 77845 USA) and IBM, SPSS version 29 (SPSS Inc., Chicago, IL, USA).

We performed univariate and multivariate Cox proportional hazards regression analysis. With the multivariate model we assessed the association between LAVi as well as other variables potentially influencing mortality and MACE. We selected relevant echocardiographic variables (together with age) for the multivariate model that demonstrated a significant association with mortality and MACE in the univariate model. These variables – age, year of enrollment, LV GLS, LV EF, LV filling pressure, moderate/severe MR, and LAVi – were included in the final multivariate model. GLS and EF showed moderate to high degree of correlation (*r* = 0.57, *p* < 0.001) [25, 26] and were divided in two separate models. Left ventricular end diastolic- and end systolic volume showed a high degree of correlation (*r* = 0.90, *p* < 0.001) and were excluded from the multivariate model. The assumption of proportional hazards was tested by using Schoenfeld residuals.

We used Kaplan-Meier survival curves to plot survival free from all-cause mortality in patients with dilated LA (LAVi > 34 mL/m^2^) and normal LA (LAVi ≤ 34 mL/m^2^). Differences between survival curves was assessed by the log-rank test.

Intra- and interobserver variability were determined by repeating the LA volume measurements from the same dataset > 1 month apart in 25 randomly selected patients and expressed by coefficient of variation.

## Results

### Demographic information and in-hospital management

Table 1 illustrates the clinical characteristics and echocardiographic data. The study encompassed a cohort of 487 patients diagnosed with AMI. Their mean age was 69 ± 12 years and 127 (26.1%) were women. Half of the patients had a history of hypertension (HT), while one fifth had diabetes mellitus (DM). Approximately three quarter of the patients experienced their first AMI. Over 90% of the patients received PCI as a part of their treatment regimen. Left anterior descending artery emerged as the most frequently treated artery. Less than one third of the patients exhibited multivessel coronary artery disease, characterised by stenosis in two or more coronary arteries. In our study, 412 patients (84.6%) underwent complete revascularisation, and 333 patients (68.4%) were found to have one-vessel disease.

**Table 1:** Demographic information and in-hospital management of patients with myocardial infarction

Variable	Individuals *n* = 487
Age, years	69 ± 12
Gender, female (%)	128 (26.2)
STEMI, n (%)	34 (7.0)
NSTEMI, n (%)	453 (93.0)
**Risk factors**	
Smoke (current and last 10 years), n (%)	286 (60.3)
Body mass index, kg/m^2^	27 ± 4
Weight, kg	
Female	75.0 ± 13.6
Male	88.1 ± 14.0
Hypertension, n (%)	247 (50.7)
Diabetes Mellitus, n (%)	102 (21.0)
Hypercholesterolemia, n (%)	136 (29.3)
Previous HF, n (%)	19 (3.9)
Previous MI, n (%)	120 (24.6)
First time MI, n (%)	367 (75.3)
Previous CABG, n (%)	33 (6.8)
SBP, mmHg	133 ± 20
DBP, mmHg	76 ± 12
Heart rate, bpm	70 ± 13
AF at admission, n (%)	27 (5.5)
**Clinical assessment**	
NYHA ≥ 2 admission, n (%)	83 (17.0)
**Echocardiography**	
LV EF, %	54 ± 9
LV GLS, %	-15.2 ± 3.2
LV EDVi, mL/m^2^	63.5 ± 21.3
LV ESVi, mL/m^2^	29.7 ± 14.9
LAVi, mL/m^2^	30.7 ± 11.8
Elevated LV filling pressure, n (%)	80 (16.6)
MR ≥ 2, n (%)	29 (6.0)
TR, n (%)	228 (69.9)
TR pressure gradient, mmHg	22 ± 10
PAPS, mmHg	31 ± 14
**Blood samples** Hs-cTnI, ng/L	3712 (223–3338)
Hs-cTnT, ng/L	1377 (160–1654)
Creatinin, µmol/L	86 ± 45
NT-proBNP, ng/L	1871 (186–1675)
**Treatment**	
PCI, n (%)	448 (92.0)
**Coronary artery with significant stenosis**	
Left main coronary artery, n (%)	39 (8.0)
Left anterior coronary artery, n (%)	263 (54.0)
Left circumflex coronary artery, n (%)	188 (38.6)
Right coronary artery, n (%)	164 (33.7)
Multivessel disease, n (%)	154 (31.6)
**Not revascularized**	
No significant stenosis, n (%)	16 (50.0)
Not suitable, n (%)	4 (12.5)
OMT, n (%)	11 (34.4)
**Medication at discharge**	
Platelet inhibitor, n (%)	487 (100)
Beta-blocker, n (%)	326 (66.9)
ACE inhibitor/ARB, n (%)	260 (54.1)
Statins, n (%)	458 (94.2)
Diuretics, n (%)	69 (14.2)

Data are expressed as numbers (%), mean ± SD or median (Interquartile Range) as appropriate

*ACE*, angiotensin-converting enzyme; *AF*, atrial fibrillation; *ARB*, angiotensin receptor blocker; *CABG*, coronary artery bypass grafting; *DBP*, diastolic blood pressure; *EDVi*, end-diastolic volume index; *EF*, ejection fraction; *ESVi*, end-systolic volume index; *GLS*, global longitudinal strain; *HF*, heart failure; *Hs-cTnI*, high-sensitivity cardiac troponin I; *Hs-cTnT*, high-sensitivity cardiac troponin T; *LAVi*, left atrial volume index; *LV*, left ventricular; *MI*, myocardial infarction; *MR*, mitral regurgitation; *NSTEMI*, non-ST-segment Elevation Myocardial Infarction; *NT-proBNP*, N-terminal pro-B-type natriuretic peptide; *NYHA*, New York Heart Association Classification; *OMT*, optimal medical therapy; *PAPS*, pulmonary artery pressure systolic; *PCI*, percutaneous coronary intervention; *SBP*, systolic blood pressure; *STEMI*, ST-segment Elevation Myocardial Infarction; *TR*, tricuspid regurgitation

There were 322 (67.1%) patients with normal LA volume (LAVi ≤ 34 mL/m^2^) and 158 (32.9%) with enlarge LA volume (LAVi > 34 mL/m^2^). Among patients who were classified in New York Heart Association Functional Classification ≥ 2, 34 (41.5%) and 48 (58.5%) had normal and dilated LA volume respectively, *p* < 0.001. Among patients who were revascularised with PCI, 299 (67.8%) and 142 (32.2%) had normal and dilated LA volume respectively, *p* = 0.261.

### Predictors of all-cause mortality and MACE

During a median follow-up period of 3.8 years (range 3.1 to 5.0 years) 153 patients (31.4%) experienced the primary clinical outcomes. Among these, a total of 50 patients (10.3%) died.

Hospital readmissions during the follow-up period included patients with myocardial infarction (*n* = 51, 10.5%), stroke (*n* = 21, 4.3%), HF (*n* = 32, 6.6%), cardiac arrest (*n* = 8, 1.6%), and new onset of AF (*n* = 47, 9.7%).

**Fig. 1:**
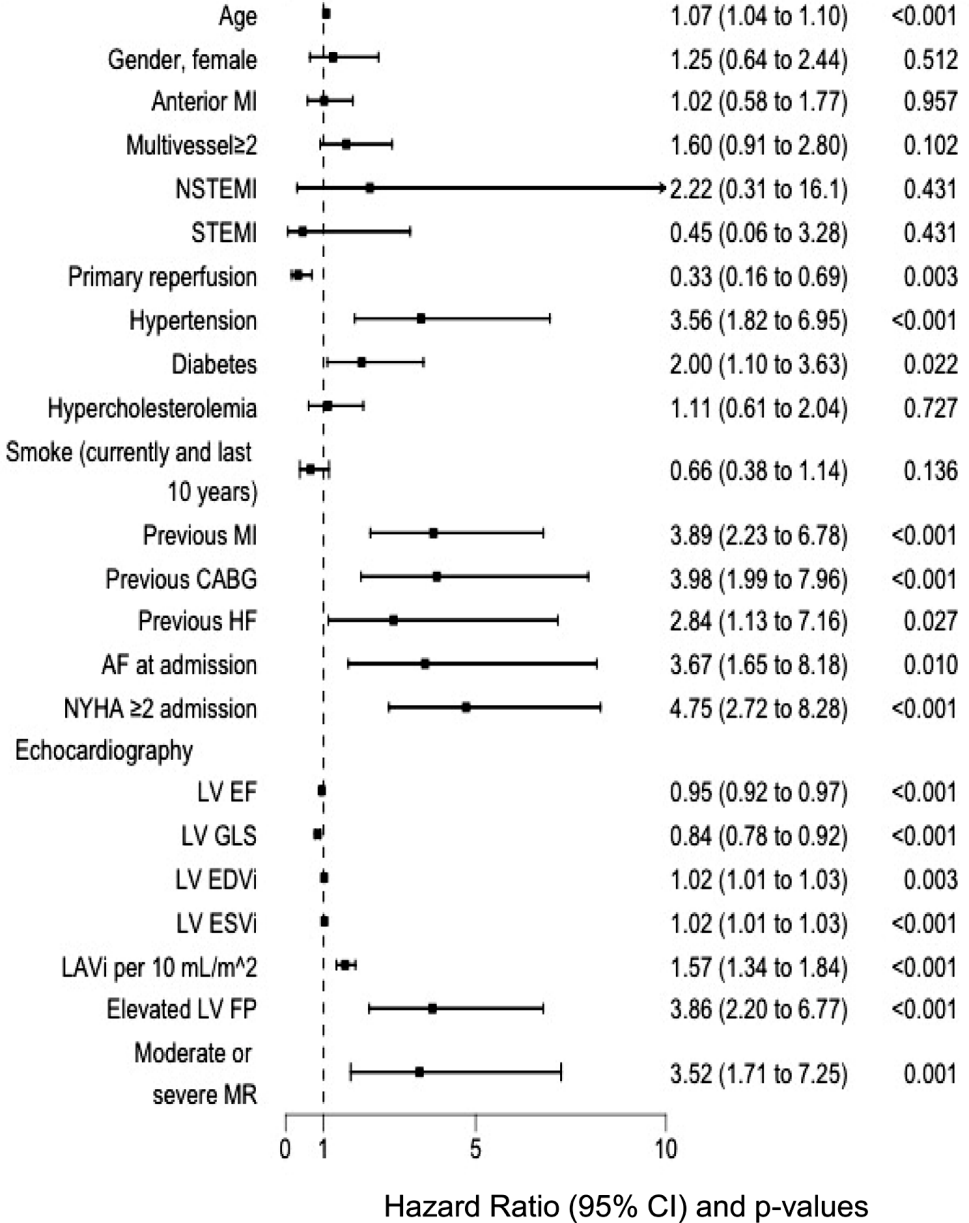
Independent risk predictors of all patients in the study by univariate Cox proportional hazards analysis. Hazard ratio (95% CI and p-value). *AF*, atrial fibrillation; *CABG*, coronary artery bypass grafting; *EDVi*, end-diastolic volume index; *EF*, ejection fraction; *ESV*, end-systolic volume index; *FP*, filling pressure; *GLS*, global longitudinal strain; *HF*, heart failure; *LAVi*, left atrial volume index; *LV*, left ventricular; *MI*, myocardial infarction; *MR*, mitral regurgitation; *NSTEMI*, non-ST-segment Elevation Myocardial Infarction; *NYHA*, New York Heart Association Classification; *STEMI*, ST-segment Elevation Myocardial Infarction

Figure 1 presents the results of univariate Cox proportional hazards analysis. A multivariate Cox proportional hazard analysis is provided in Table 2. Notably, the analysis revealed that for every 10 mL/m² increase in LAVi the hazard ratio (HR) was 1.21 (95% CI 1.05–1.38) (*p* = 0.006). An equivalent finding for LAVi was reflected when LV GLS was replaced by LV EF, yielding a HR of 1.22 (95% CI 1.07–1.39), *p* = 0.003. Additionally, GLS and EF were both independently significant in their respective models (HR 0.92 [95% CI 0.87–0.97], *p* = 0.004 and HR 0.97 [0.95–0.99], *p* = 0.004). LA reservoir strain was significant as a predictor of all-cause mortality and MACE in the univariate Cox regression model with an HR of 0.94 (95% CI: 0.92–0.96, *p* < 0.001). We used Schoenfeld residuals to test proportionality for each of the variables used in the univariate and multivariate Cox model, which was visually proven with flat curves.

**Table 2:** Potential predictors^a^ of primary clinical outcomes^b^ in a multivariate Cox proportional hazard analysis

Characteristics	Hazard Ratio	95% CI	*P*-value
Age (years)	1.02	1.01–1.04	0.001
Year of enrollment (number)	0.65	0.58–0.72	< 0.001
LV GLS (%)	0.92	0.87–0.97	0.004
Elevated LV filling pressure	1.26	0.83–1.91	0.286
Moderate/severe MR	1.38	0.80–2.39	0.250
LAVi 10 mL/m^2^	1.21	1.05–1.38	0.006

Data are expressed as hazard ratios, their 95% CI and p-values

^a^Tested variables were age, LV GLS, elevated LV FP, moderate/severe MR and LAVi. ^b^Primary clinical outcomes were available for 487 patients during follow-up time

*GLS*, global longitudinal strain; *LA*, left atrial; *LAVi*, left atrial volume index; *LV*, left ventricular; *MR*, mitral regurgitation

**Fig. 2:**
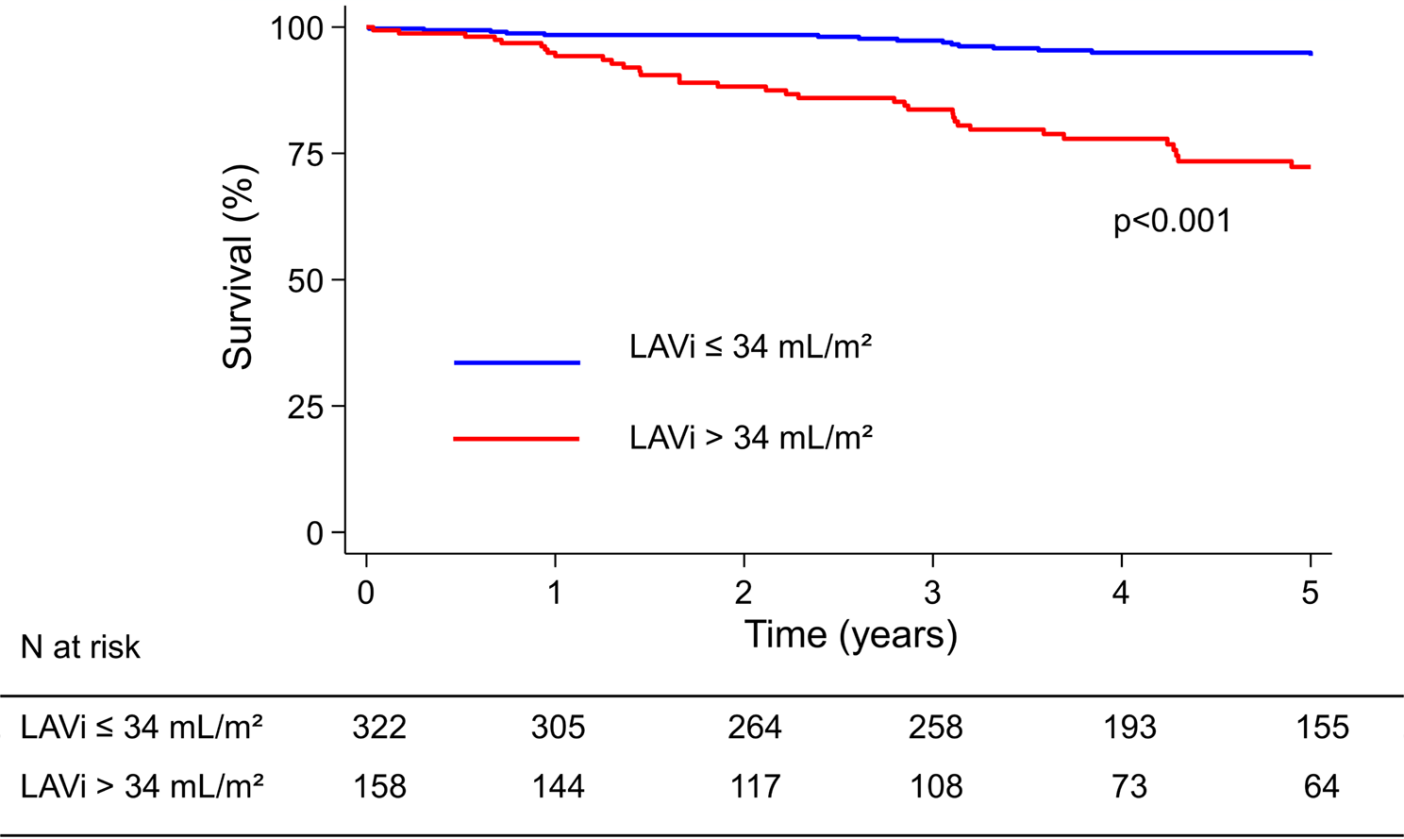
Kaplan-Meier survival curves. Blue curve shows patients with LAVi ≤ 34 mL/m^2^ and red curve shows patients with LAVi > 34 mL/m^2^. The log-rank test is used to compare survival curves (*p* < 0.001). N at risk table gives information about how many patients at risk at a given time for each group LAVi ≤ 34 mL/m^2^ and > 34 mL/m^2^. *LAVi*, indexed left atrial volume; *N*, number

**Fig. 3:**
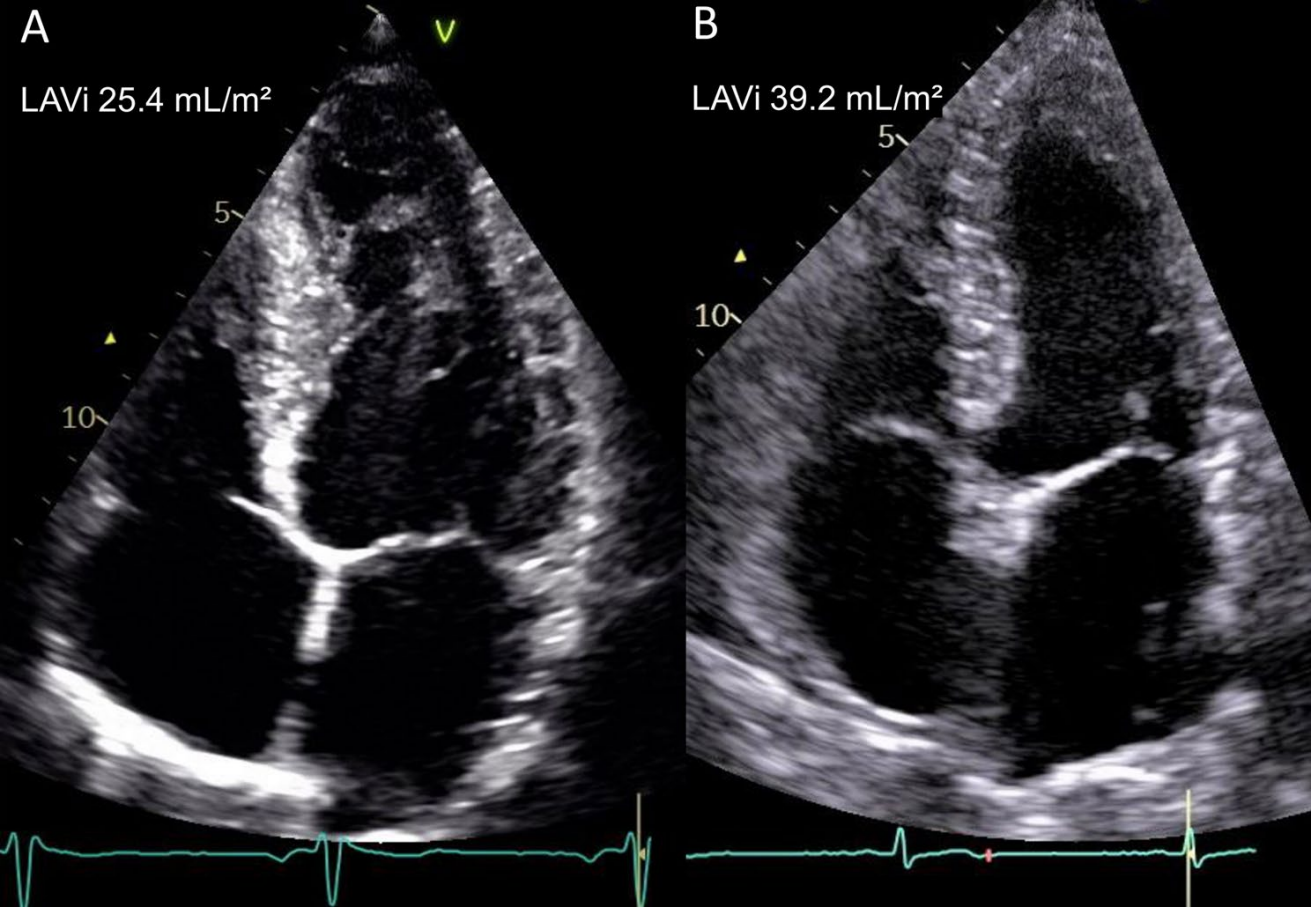
Echocardiographic images of patients with AMI. The image to the left (**A**) shows a patient with normal left atrial volume who survived the study period whereas the image to the right (**B**) shows a patient with dilated left atrial volume who died

AMI, acute myocardial infarction; LAVi, indexed left atrial volume.

The deceased patients had higher LAVi compared to survivors (40.0 ± 12.9 mL/m^2^ vs. 29.7 ± 11.2 mL/m^2^, *p* < 0.001). As demonstrated in Figs. 2 and 3 patients with LAVi > 34 mL/m² exhibited poorer survival compared to those with LAVi ≤ 34 mL/m² (*p* < 0.001).

**Fig. 4:**
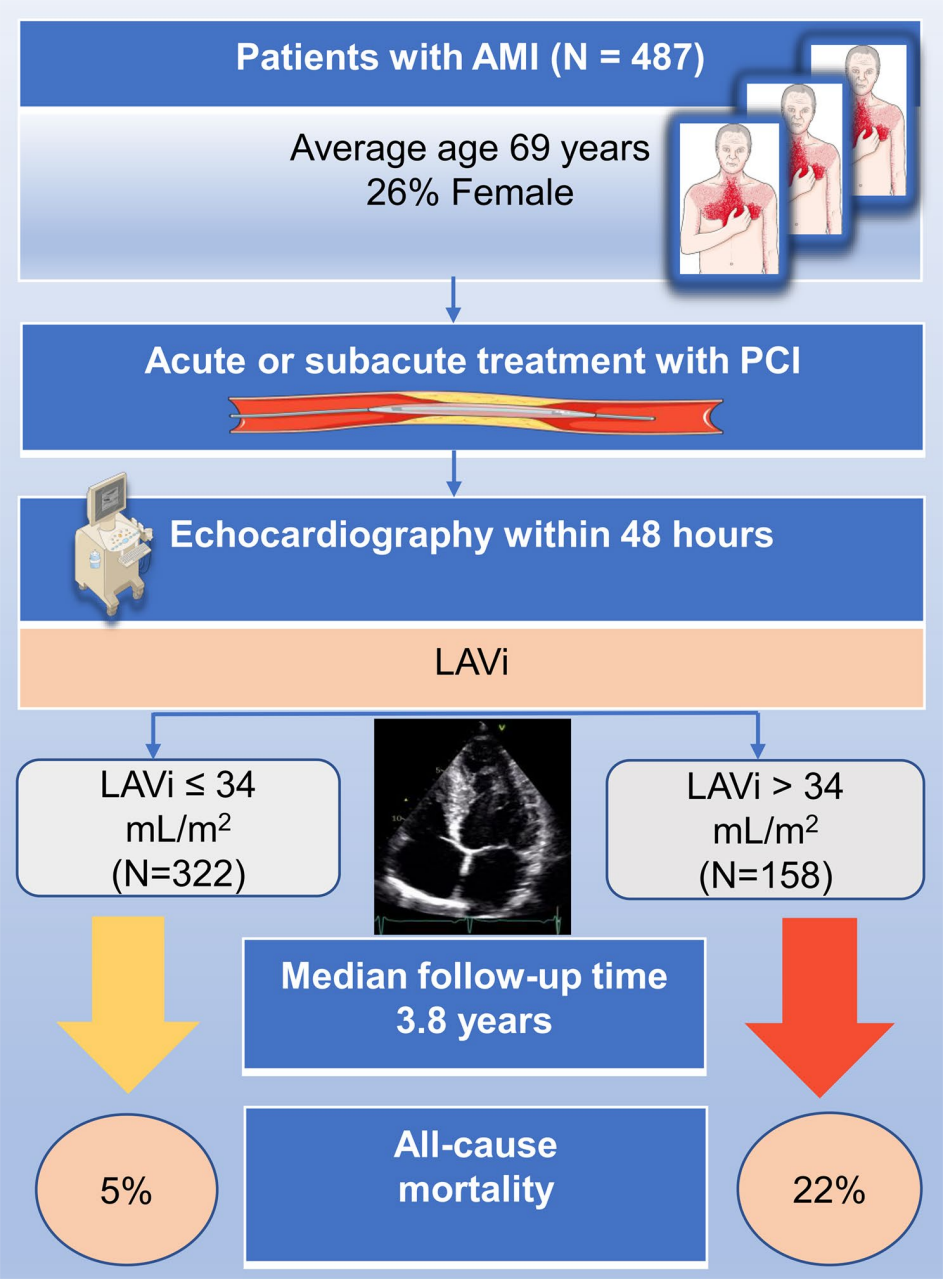
Dilated LA predicts increased mortality in patients with AMI. Summary of the study that shows that AMI patients with dilated LA have significant higher risk of death compared to those without dilated LA. *AMI*, acute myocardial infarction; *LA*, left atrium; *LAVi*, indexed left atrial volume; *PCI*, percutaneous coronary intervention

In patients with an elevated LAVi (> 34 mL/m^2^), a significant proportion of 22.2% (35 patients) experienced mortality. In contrast, among those with a normal LAVi (≤ 34 mL/m^2^), only 4.7% (15 patients) faced the same outcome (Fig. 4).

Among the patients who were admitted with AF (*n* = 27, 5.5%), a total of 16 patients (10.5%) experienced the primary clinical outcomes. It is noteworthy that out of these 16 patients, 15 of them had an elevated LAVi (> 34 mL/m^2^), *p* < 0.001.

Out of the total number of patients who had moderate/severe MR (*n* = 29, 6.0%), only 21 patients (6.0%) experienced the primary clinical outcomes. Notably, out of these 21 patients, 19 of them had an elevated LAVi (> 34 mL/m2), *p* < 0.001.

### The impact of atrial fibrillation and mitral regurgitation on left atrial volume

We recorded patients with AF at admission (*n* = 27, 5.5%) and they had increased LAVi compared to those who not had AF at admission (*n* = 453, 94.4%), 45.5 ± 14.3 vs. 29.9 ± 11.0 mL/m^2^ respectively, *p* < 0.001.

Patients with moderate/severe MR (*n* = 29, 6.0%) had elevated LAVi compared to those who had non to mild mitral regurgitation (*n* = 455, 94.0%), 46.3 ± 13.6 vs. 29.8 ± 10.9 mL/m^2^ respectively, *p* < 0.001.

### Left ventricular and atrial function and characteristics

The relationship between LAVi and LV function are shown in Table 3. Notably, patients exhibiting moderate and severely abnormal LV EF displayed significantly larger LAVi compared to those with normal LV function (*p* < 0.001).

Among the subset of patients with LV EF < 45% who died (*n* = 14), we found only one patient with normal LAVi whereas 13 patients had dilated LA (*p* = 0.028).

Inter- and intraobserver variability repeating the LA volume measurements was excellent with intraclass correlation coefficients at 0.986 and 0.984, respectively (both *p* < 0.001) (Additional file 2).

**Table 3:** Left ventricular ejection fractions impact on left atrial volume

LV EF	LAVi per mL/m^2^	*p*-value
Normal (≥ 53%), *n* = 326 (67.1%)	29.6 ± 10.2	
Mildly abnormal (41–52%), *n* = 119 (26.7%)	29.4 ± 12.0	0.885
Moderately abnormal (30–40%), *n* = 31 (8.7%)	42.7 ± 14.6	< 0.001
Severely abnormal (< 30%), *n* = 11 (3.3%)	44.6 ± 18.9	< 0.001

Data are expressed as numbers (%) and mean ± SD

Normal LV EF serves as the reference group. Mildly-, moderately- and severely abnormal LV EF compares each to the reference group

*EF*, ejection fraction; *LAVi*, indexed left atrial volume; *LV*, left ventricular

### Sex differences

There were no significant differences in primary outcomes in those with elevated LA volume when comparing the sexes. There were 41 women (32.2%) and 112 men (31.1%) who reached the primary endpoints (*p* = 0.807), of these, 18 women and 57 men had elevated LAVi > 34 mL/m^2^, *p* = 0.491.

## Discussion

The current study is the first comprehensive evaluation of LA volume as a risk indicator following up-to-date treatment of AMI-patients. The pivotal discovery is the compelling association between augmented LA volume and subsequent all-cause mortality and MACE over a median span of 3.8 years. Among the spectrum of variables examined, it became clear that LA volume, along with LV GLS and LV EF, stood out as the most reliable and consistent echocardiographic indicators of adverse outcome in this study. The adverse outcome reaffirmed the importance of LA volume as a critical parameter for evaluating cardiovascular risk and determining patient prognosis following AMI, even in in the context of contemporary treatment.

### Mortality after AMI

Treatment strategies for AMI have evolved over the past decades. Early angiography increased from 9% in 1995 to 60% in 2015, coupled with a rise in PCI during the initial hospital stay, from 13 to 67%. These changes resulted in a reduction in 6-month mortality from 17 to 6%. [7, 9] In the early 2000s, DAPT (aspirin + clopidogrel) emerged as the standard secondary prevention therapy in AMI patients, [10–12] a practice uniformly adopted in our study. While DES were not universally accepted over BMS in 2007, the guidelines began favoring DES over BMS in AMI patients in 2012 and 2015. [13, 27] Our patients predominantly received treatment with DES, aligning with current guidelines. Our study, compared to the meta-analysis by Ahmeti et al. in 2021 [28], provides valuable insights into contemporary AMI management, emphasising early revascularisation, DAPT, and the widespread use of DES.

Patients who have recovered from AMI face a heightened risk of recurrent events and premature mortality. [11, 29] Two decades ago, Møller et al. [1] introduced the importance of LA volume as a predictor of post-AMI survival. Their retrospective study unveiled that an enlarged LA volume stood as a robust independent predictor of all-cause mortality over a 15-month follow-up period. Subsequently, another study [2] conducted a five-year prospective analysis on AMI patients and validated that LA volume retained its status as a noteworthy predictor of post-AMI mortality. However, the evolution in AMI treatment strategies since these studies has resulted in a substantial reduction in six-month mortality, from 17 to 6%. [7, 9] Our study shows, however, that the predictive potency of LA volume endures as a crucial marker for all-cause mortality and MACE even among the novel and effective approaches to in-hospital and preventive medical interventions for AMI patients over the past two decades.

### LA dilatation as a marker of disease

LA volume appeared as a stable parameter, despite updated treatment strategies. It adapted the influences of elevated LV filling pressures from preexisting cardiovascular conditions as well as dynamic alteration induced by AMI. [30] LV diastolic dysfunction is a well-established marker of risk stratification in patients with AMI. [31] The most important determinators for diastolic dysfunction are age, HT, DM and previous infarction, all factors demonstrated to indicate increased mortality in the current study. LA volume, however, reflects the influence of these factors and additional pathological processes, such as diastolic dysfunction secondary to prior myocardial infarction, the prolonged presence and severity of MR, and the occurrence of AF. Several of these factors listed above could have existed prior to the acute infarction and continued to exert importance after the event. Therefore, improved treatment of the AMI may have limited impact in other diseases also important for the prognosis, leaving the increased LA volume as a marker of chronic disease rather than a direct consequence of AMI.

### LV function and LA volume

Our findings emphasise the intricate interplay and dynamic relationship between LV function and LA volume as we observed both LV GLS and LV EF and LAVi significance in the multivariate Cox regression model. Patients with HF are known to be in an increased risk of developing high LV filling pressure and other pathological conditions, resulting in an enlarged LA. The significance of LV GLS, LV EF and LAVi in our multivariate model highlights the interconnection of these parameters, suggesting that changes in LV function can influence LA volume and vice versa.

Despite the evolving landscape of in-hospital and prophylactic treatments for AMI, which have contributed to a reduction in overall cardiovascular mortality, our study’s main contribution lies in demonstrating that the prognostic value of LA volume remains unchanged. This underscores that while new treatments for AMI may improve outcomes, they may not fully address pre-existing comorbidities. Thus, LA volume continues to serve as a valuable prognostic indicator, emphasising the need for comprehensive management strategies that address both acute and chronic aspects of cardiovascular disease.

### Other cardiovascular diseases and LA

Various cardiovascular diseases aside from coronary artery diseases, show a strong correlation between LA size and prognosis. A registry study of 788 patients with MR due to flail leaflets found that an LA diameter ≥ 55 mm was associated with increased mortality under medical treatment, regardless of LV dysfunction. In contrast, an LA diameter ≥ 55 mm did not affect postoperative outcomes in patients who underwent mitral surgery. Furthermore, mitral surgery was found to have a greater survival benefit in patients with an LA diameter ≥ 55 mm compared to those with a LA diameter < 55 mm. [32] Recently, a multicenter registry study including 554 individuals with moderate to severe aortic regurgitation and bicuspid aortic valve, showed that LA dilatation independently predicted reduced event-free survival. [33]

### Clinical importance

Our findings may have two important clinical implications. Firstly, LA volume should be incorporated into the assessment of AMI patients, as it imparts substantial additional prognostic insights. Secondly, an enlarged LA volume could serve as an indicator necessitating intensified treatment of underlying conditions such as hypertension and diabetes, both of which significantly influence prognosis. This should again lead to more thorough follow-up by a cardiologist.

### Limitation

The study underlined the significance of assessing LA dilatation through early echocardiography, conducted within the initial 48 h of admission for patients with AMI. However, it is important to acknowledge that at this early stage of LV dysfunction, the full remodeling process after the myocardial infarction is not done. Therefore, our findings might not be directly extrapolated to studies performed at a later stage.

Another consideration pertains to the intrinsic three-dimensional nature of the LA. In our study we measured LA volume in a two-dimensional way, from both two- and four chamber view. Three-dimensional imaging might therefore be an even more accurate method of defining LA volume and function.

Assessments of LA volumes are generally more robust by 3D compared to 2D echocardiography. [17] Our study, however, was planned and implemented as a 2D echocardiographic study. Although LA minimum volume by 3D echocardiography has shown significant relevance [34–36], LA maximum volume is the more commonly utilised and well-established measurement [1, 2, 19].

The results in our study could be influenced by the non-consecutive enrollment process and the availability of physicians during daytime hours. This potentially introduces selection bias, affecting the generalisability of our findings. Additionally, the high proportion of revascularised patients (92.0%) is due to the inclusion of myocardial infarction type 1 cases, with type 2 cases excluded. This distinction is critical when interpreting outcomes related to revascularisation and warrants caution in extrapolating our results to broader populations.

The inclusion of patients with AF at admission and the acknowledgment that some with paroxysmal or subclinical AF might have been missed is relevant. AF is known to be associated with increased mortality risk in AMI patients, but our study focused on left atrial volume as an independent predictor, regardless of AF status. [37]

## Conclusion

Our study demonstrated that LA volume enlargement remained as an independent echocardiographic predictor of adverse outcome after AMI, despite notable advancements in treating patients with AMI over the past two decades. Our findings underscore the importance of comprehensive echocardiographic evaluation in post-AMI care. The persistence of LA volume as a significant predictor highlights its potential utility as a readily accessible and informative tool for guiding future surveillance and therapeutic decisions in patients recovering from AMI.

## Electronic supplementary material

Below is the link to the electronic supplementary material.

Supplementary Material 1: Title: Left ventricular filling pressure. Description: Non-invasive measure of left ventricular filling pressure

Supplementary Material 2: Title: Bland-Altman plots. Description: Bland-Altman plots comparing two sets of measurements of LAVi conducted by the same operator (left) and by two different operators (right). The horizontal axis shows the mean of both measurements, and the vertical axis shows the difference between both measurements drawn. The plots show the spread between the results of both sets of measurements which increases with higher mean values. LAVi, indexed left atrial volume.

## Data Availability

No datasets were generated or analysed during the current study.

## Abbreviations

AF: Atrial fibrillation
AMI: Acute myocardial infarction
BMS: Bare-metal stents
CABG: Coronary artery bypass grafting
DAPT: Duel antiplatelet therapy
DES: Drug-eluting stents
EF: Ejection fraction
GLS: Global longitudinal strain
LA: Left atrium(ial)
LAVi: Indexed left atrial volume
LV: Left ventricular(cle)
MACE: Major adverse cardiac events
MR: Mitral regurgitation
PCI: Percutaneous coronary intervention
