# Supplementary material for: Left atrial volume assessed by echocardiography identifies patients with high risk of adverse outcome after acute myocardial infarction
Source: Echo Res Pract. 2024 Oct 21;11:24. doi: 10.1186/s44156-024-00060-1 (PMC11492485; doi:10.1186/s44156-024-00060-1)
Supplement: Supplementary file 1 — Supplementary Material 1: Title: Left ventricular filling pressure. Description: Non-invasive measure of left ventricular filling pressure [file 44156_2024_60_MOESM1_ESM.pdf]

**Non-invasive measure of left ventricular filling pressure**

The evaluation of left ventricular (LV) filling pressure was conducted in accordance with the 2022 European Association of Cardiovascular Imaging consensus document, (1) incorporating a combination of five echocardiographic parameters. (2) This algorithm includes E/A, E/e', tricuspid regurgitation (TR) velocity, indexed left atrial (LA) volume (LAVi) and LA reservoir strain, each with predefined cut-off values.

If mitral E was <0.5 m/s and E/A was ≤0.8, LV filling pressure was regarded normal or low. Whereas E/A ≥2 indicated elevated LV filling pressure.

When the mitral E/A was between 0.8 and 2.0 additional criteria were needed to assess LV filling pressure. This included average E/e' >14, peak TR velocity >2.8 m/s, and LAVi >34 mL/m2. If ≥2 of these parameters were above the cut-off, LV filling pressure was regarded elevated. If ≥2 parameters were below the cut-off, LV filling pressure was regarded normal. (E/e' was calculated using the average of septal and lateral e'. When only septal or lateral e' were used, the recommended cutoffs for identifying elevated LV filling pressure, were 15 and 13, respectively.)

LA reservoir strain was used when only two of three criteria were available and those two were conflicting (one positive and one negative). (1) LV filling pressure was assumed elevated when LA reservoir strain <18%. (2)


1. Smiseth OA, Morris DA, Cardim N, Cikes M, Delgado V, Donal E, et al. Multimodality imaging in patients with heart failure and preserved ejection fraction: an expert consensus document of the European Association of Cardiovascular Imaging. Eur Heart J Cardiovasc Imaging. 2022;23(2):e34-e61.

2. Inoue K, Khan FH, Remme EW, Ohte N, García-Izquierdo E, Chetrit M, et al. Determinants of left atrial reservoir and pump strain and use of atrial strain for evaluation of left ventricular filling pressure. Eur Heart J Cardiovasc Imaging. 2021;23(1):61-70.
