# Supplementary figures and images for: Left atrial volume assessed by echocardiography identifies patients with high risk of adverse outcome after acute myocardial infarction
Source: Echo Res Pract. 2024 Oct 21;11:24. doi: 10.1186/s44156-024-00060-1 (PMC11492485; doi:10.1186/s44156-024-00060-1)

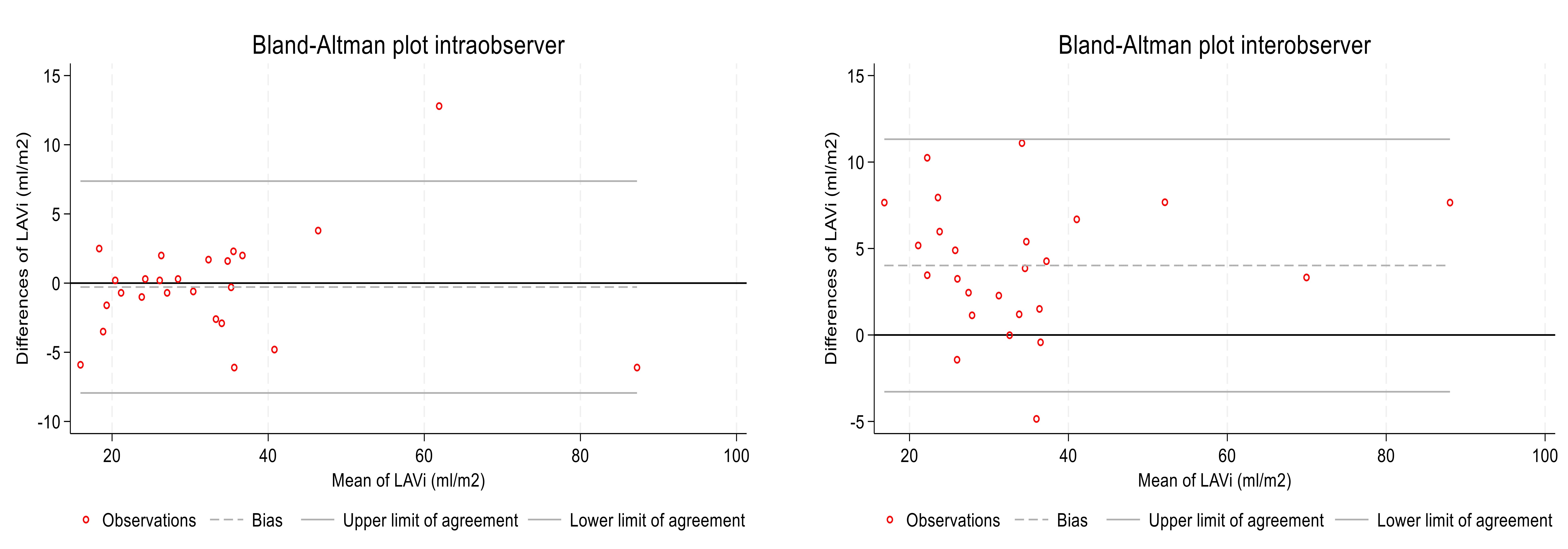

Supplement: Supplementary file 2 — Supplementary Material 2: Title: Bland-Altman plots. Description: Bland-Altman plots comparing two sets of measurements of LAVi conducted by the same operator (left) and by two different operators (right). The horizontal axis shows the mean of both measurements, and the vertical axis shows the difference between both measurements drawn. The plots show the spread between the results of both sets of measurements which increases with higher mean values. LAVi, indexed left atrial volume. [file 44156_2024_60_MOESM2_ESM.jpg]
